# Looking Deep Into the Palpitation – Pheochromocytoma Presenting as Sinus Node Dysfunction

**DOI:** 10.7759/cureus.17151

**Published:** 2021-08-13

**Authors:** Abhinav Shrivastava, Ranjit K Nath, Puneet Aggarwal

**Affiliations:** 1 Cardiology, Atal Bihari Vajpayee Institute of Medical Sciences and Dr. Ram Manohar Lohia Hospital, New Delhi, IND

**Keywords:** secondary hypertension, adrenal pheochromocytoma, bradyarrythmia, junctional rhythm, nodal escape

## Abstract

A 23-year-old male came to the outpatient department with a history of intermittent palpitations and headaches for the past six to eight months. He was diagnosed with hypertension and had a junctional rhythm on an electrocardiogram (ECG). On further workup for his hypertension, he was found to have elevated levels of serum metanephrines and computed tomography (CT) and positron emission tomography (PET) scan revealed pheochromocytoma. He was subsequently operated upon and his arrhythmia subsided after surgery. We discuss our approach to this scenario, which leads us to a rather rare cause of sinus node dysfunction.

## Introduction

Pheochromocytomas are rare tumors reported in 0.2% of hypertensive patients, and if unidentified, can lead to fatal outcomes [1]. Palpitations are usually included in the classic triad of pheochromocytoma along with hypertension and episodic headaches. However, most patients present with varied symptoms that are often missed, making the diagnosis more challenging. Palpitation in pheochromocytoma may either present alone or as a component of the triad and indicates an erratic electrical activity in the heart from the catecholaminergic excess. The usual causes of palpitations in this patient group are tachyarrhythmias and may manifest as sinus tachycardia to supraventricular tachycardia or ventricular tachycardias. Bradyarrhythmia and sinus node dysfunction are uncommon manifestations of this condition and have been rarely reported in the literature [2-6]. We present a patient with a unique presentation of junctional rhythm without tachycardia, as the predominant rhythm in a patient with pheochromocytoma.

## Case presentation

A 23-year-old male came to the outpatient department with a history of intermittent palpitations for the past six to eight months. He also complained of episodic headaches during the same period, which he attributed to studying and social stressors. He did not have chest or abdominal pain, dyspnea, leg swelling, leg cramps, or sleep disturbance. He confided no known addictions and gave no contributing family history. He was not a known hypertensive and was not taking any medications for his symptoms. On examination, his body mass index was 19 kg/m^2^. He was having a pulse rate of 70/minutes, which was regular in rhythm and normal in volume with no special character, and blood pressure of 150/100 mmHg in the right arm and 156/102 mmHg in the left arm with normally palpable femoral arteries. He appeared anxious but had a normal general physical build. Fundus examination showed mild arteriolar narrowing, with sharp disc margins. Jugular venous pressure was not elevated. No carotid bruits or neck swelling was detected. Auscultation noted irregular heart sounds with no murmur. The irregularity in heart sound was appreciable only on long auscultation. Other systemic examinations were within normal limits.

**Figure 1:**
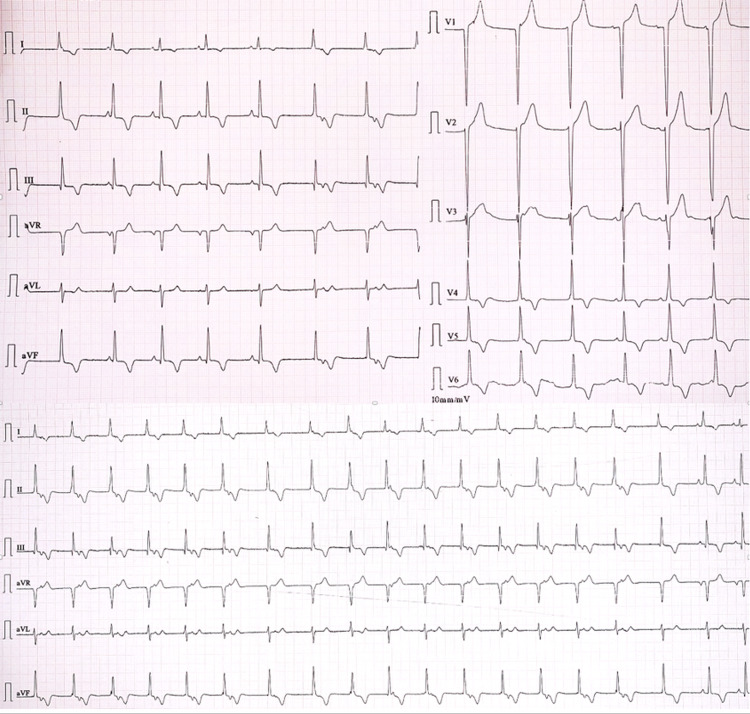
Electrocardiogram showcasing progressive sinus rhythm slowing with periods of junctional rhythm with retrograde atrial conduction. Diffuse T-wave inversions in leads I, II, III, aVF, V4, V5, and V6.

**Figure 2:**
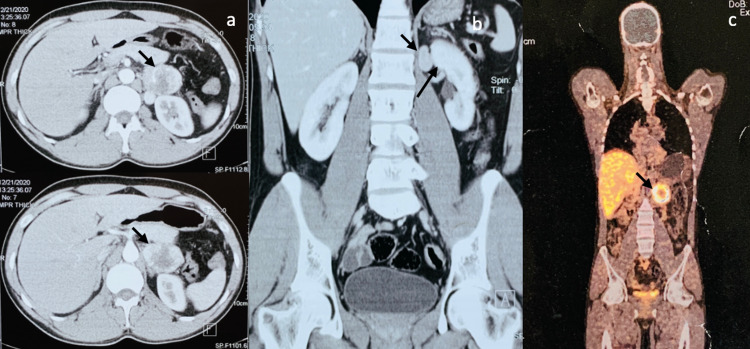
(a) and (b) Computerized tomography scan of the abdomen was ordered, which revealed a well-defined (35 x 46 x 55 mm) mass lesion in left peri-renal space with heterogenous intense arterial enhancement in close proximity to another similar homogenous lesion (20 x 17 x 32 mm), not seen separately from the left adrenal gland suggesting adrenal as well as extra-renal pheochromocytoma/paraganglioma. (c) Ga68-DOTANOC whole-body PET-CT similarly described the mass lesions expressing somatostatin receptors (SSTR). No other lesion was present elsewhere. DOTANOC - DOTA-(Nal3)-octreotide; PET-CT - positron emission tomography-computed tomography.

**Figure 3:**
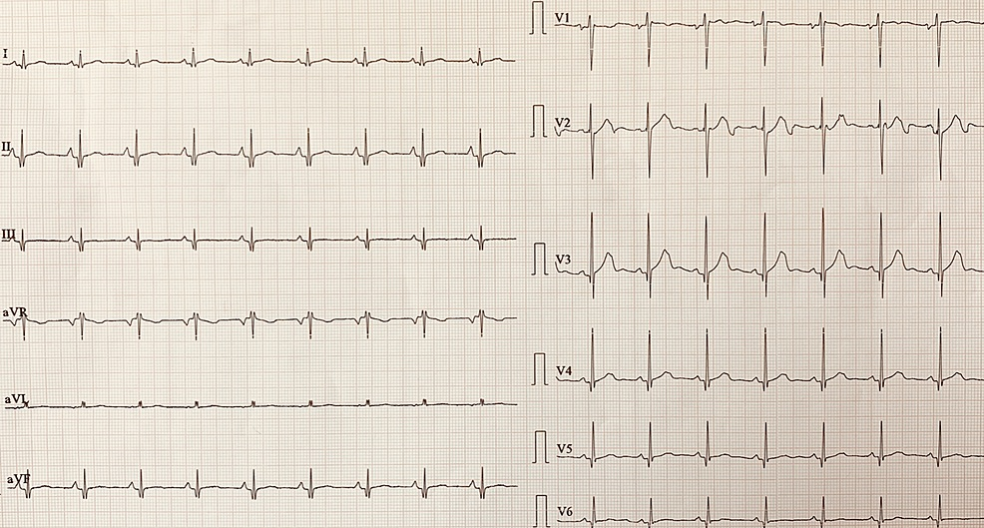
Electrocardiogram showing normal sinus rhythm post-surgery.

Electrocardiograph (ECG) (Figure 1) revealed a junctional rhythm (JR) with intermittent sinus beats and diffuse T-wave inversions in leads I, II, III, aVF, V4, V5, and V6. Transthoracic echocardiogram was suggestive of left ventricular hypertrophy with normal valvular, systolic, and diastolic functions. There was no evidence of coarctation of the aorta or anomalies in venous return. A 48-hour Holter ECG monitoring was issued and captured symptoms of palpitations predominantly associated with junctional rhythm with rates between 50 and 80 beats per minute. The kidney duplex ultrasound scan did not show any evidence of renal artery stenosis. The full blood counts, kidney and thyroid function tests, and serum electrolytes were within the normal range. As part of the etiological search of this hypertension associated with palpitations, free plasma metanephrines were done, which revealed elevated levels of serum normetanephrine, 7480 ng/L (normal range: 20.10-135.4 ng/L). Serum metanephrines, 14.2 ng/L (7.9-88.7 ng/L), and serum 3-methoxytyramine, 6.14 ng/L (<18.4 ng/L), were in normal range. Given the abnormal results, a contrast-enhanced computerized tomography (CT) scan of the abdomen was ordered, which revealed a well-defined (35 x 46 x 55 mm) mass lesion in left peri-renal space with heterogenous intense arterial enhancement in close proximity to another similar homogenous lesion (20 x 17 x 32 mm), not seen separately from the left adrenal gland suggesting adrenal as well as extra-renal pheochromocytoma/paraganglioma (Figures 2a, 2b). A Ga68-DOTA-(Nal3)-octreotide (DOTANOC) whole-body positron emission tomography-computed tomography (PET-CT) revealed similarly described mass lesions expressing somatostatin receptors (SSTR) (Figure 2c). The lesion was non-malignant and not present elsewhere in the body. The patient was subsequently operated upon and his pheochromocytoma was removed. Post-surgery, his ECG after 24 hours and 24-hour Holter ECG monitoring exhibited a normal sinus rhythm (Figure 3). The histopathology of tissue also confirmed pheochromocytoma with tissue composed of intermediate to large cells arranged in a trabecular, solid, and alveolar pattern, which were surrounded by a capillary-rich framework.

## Discussion

Pheochromocytoma is a rare but important cause of secondary hypertension, reported in 0.2% of hypertensive patients, and the majority of patients (50%-70%) present with palpitations and headaches [1]. Stimulation of predominantly α1 and β1 receptors by the excess of norepinephrine results in vasoconstriction causing hypertension, and positive chronotropic, dromotropic, and inotropic effects. Nevertheless, bradycardia has been reported and seems to occur in up to 10% of patients during the illness [2]. Disturbances of the autonomic nervous system have been known to influence the sinus node function. Noradrenaline infusion in healthy subjects causes baroreceptor mediated vagal activation, resulting in accentuated sympathovagal interaction. Beat-to-beat fluctuations in R-R intervals in these patients depend on the summation and timing of the opposing effects of norepinephrine and acetylcholine on the sinus node. The lack of tachycardia in these patients suggests that the reflex vagal response overrides or masks the stimulating effect of the circulating catecholamines. Adrenergic receptor desensitization can also happen over a period of time and contribute to sinus node dysfunction. Sinus node dysfunction with intermittent sinus arrest and atrioventricular (AV) nodal escape rhythm has been described although very rarely in literature [2-6]. In these instances, the diagnosis of pheochromocytoma as the cause of sinus arrest or atrioventricular dissociation was frequently delayed, even resulting in pacemaker implantation in some cases [5,7]. As pheochromocytoma is a disease with ‘multiple faces’ due to its highly variable clinical presentation, it becomes pertinent to recognize it in its presentation as a palpitation. Hypertension was one of the presenting features in our patient, which was an important leading point towards the diagnosis. However, this may not always be the case as the predominantly epinephrine secreting tumor often presents with palpitations where hypertension is not usually the presenting feature.

## Conclusions

Our patient had a unique presentation of junctional rhythm without tachycardia, as the predominant rhythm. After resection of the tumor, the arrhythmias resolved in all cases previously reported, highlighting its transient nature and it underlines that sinus node dysfunction with intermittent sinus arrest and AV-nodal escape rhythm is a potential early manifestation of pheochromocytoma. Adequate recognition and treatment of the same can reverse the rhythm abnormality and may avoid unnecessary procedures such as electrophysiological testing and pacemaker implantation.
